# Celecoxib, indomethacin, and ibuprofen prevent 6-hydroxydopamine-induced PC12 cell death through the inhibition of NFκB and SAPK/JNK pathways

**DOI:** 10.22038/IJBMS.2019.34011.8091

**Published:** 2019-05

**Authors:** Elham Ramazani, Zahra Tayarani-Najaran, Masoud Fereidoni

**Affiliations:** 1 Department of Biology, Faculty of Science, Ferdowsi University of Mashhad, Mashhad, Iran; 2 Biotechnology Research Center, Pharmaceutical Technology Institute, Mashhad University of Medical Sciences, Mashhad, Iran; 3 Department of Pharmacodynamics and Toxicology, School of Pharmacy, Mashhad University of Medical Sciences, Mashhad, Iran

**Keywords:** Apoptosis, Glutathione, NSAIDs, Parkinson's disease, PC12 cells, ROS

## Abstract

**Objective(s):**

The possible action of nonsteroidal anti-inflammatory drugs (NSAIDs) in the reduction of reactive oxygen species (ROS) and also as anti-apoptotic agents may suggest them as putative agents for the treatment of neurodegenerative diseases. This study was designed to explore some pathways alterations induced by NSAIDs following 6-hydroxydopamine (6-OHDA)-induced cell death in PC12 cells as an in vitro model of Parkinson's disease (PD) and to compare the effects of celecoxib, indomethacin and ibuprofen.

**Materials and Methods:**

The cell viability, ROS content, glutathione (GSH) level, and apoptosis were measured using resazurin, dichlorofluorescein diacetate (DCFH-DA), 5,5′-dithiobis-2-nitrobenzoic acid (DTNB), propidium iodide (PI) and flowcytometry, real-time PCR and western blot.

**Results:**

Based on the results, pretreatment with celecoxib, indomethacin and ibuprofen for 24 hr significantly induced concentration and time-dependent protection against 6-OHDA-induced PC12 cell death. Cell viability (*P*<0.001), GSH level (*P*<0.01) and cytoplasmic content of nuclear factor kappa B (NFκB) (*P*<0.01) were increased, also ROS content (*P*<0.001) and apoptosis biomarkers such as the cleaved caspase-3 (*P*<0.001), Bax (*P*<0.01), phospho- stress-activated protein kinases / c-Jun N-terminal kinases (P-SAPK/JNK) (*P*<0.01) and cleaved poly ADP ribose polymerase (PARP) (*P*<0.001) protein levels were all decreased after pretreatment of cells with NSAIDs in 6-OHDA-induced PC12 cells.

**Conclusion:**

It is suggested that NFκB and SAPK/JNK pathways have an important role in 6-OHDA-induced cell injury. Overall, it seems that pretreatment with NSAIDs protect dopaminergic cells and may have the potential to slow the progression of PD.

## Introduction

Parkinson’s disease (PD) is one of the most common neurodegenerative diseases, which affects elder patients with the incidence of 1% up to 4% over 80 years old (1). The environmental factors are the most common risk factor for Parkinson’s disease, while hereditary determinants have minor role for disease (2). PD affected patients suffer from motor, non-motor and cognitive symptoms (3). 

Nonsteroidal anti-inflammatory drugs (NSAIDs) can inhibit the activity of cyclooxygenase 1 (COX-1) and COX-2 isoforms, the enzymes that run the biosynthesis pathway of prostaglandins (4). COX-1 is motivated by the body and creates mediators such as thromboxane A2 (TXA_2_) and TXB2, which are used for basic housekeeping throughout the body for normal function such as regulation of gastric acid and kidney water excretion. COX-2 is stimulated by inflammatory cytokines and growth factors and produces prostaglandins (such as PGE_2_ and PGI_2_), which induce pain signals and inflammation responses. As a result, it is important to regulate inflammation without interrupting the normal body functions (5). 

Based on the inhibition selectivity of COX-2, NSAIDs are divided into four groups; >50, 5 to 50, and <5 inhibition selectivity for COX-2 and NSAIDs with weak inhibition of COX-1 and COX-2 (6). In the new classification, the two later groups are introduced as COX-1 selective and non-selective (7). Besides oxidative stress, inflammation has been shown to promote cell damage and apoptosis induction in neurodegenerative disorders such as PD (8). Although there are some evidences that show NSAIDs suppress the growth of cancer cells in prostaglandin-dependent and prostaglandin-independent manner, they disrupt the mitochondrial function and increase the reactive oxygen species (ROS)-induced apoptosis (9); but, particular selective COX-2 inhibitors scavenge ROS and protect cells against apoptosis (10). 

6-Hydroxydopamine (6-OHDA) is a neurotoxin, which is widely used to induce cell injury. It is suitable for both *in vitro* and *in vivo* models preparation of PD. 6-OHDA generates ROS-induced apoptosis through oxidative damage to proteins, lipids and DNA (11). The new therapeutic aspect of NSAIDs is to introduce them as a potent antioxidant with wide spectrum of application (10). The homogeneity and the accessibility to the mRNA library, similar action to dopaminergic neurons, specific response to 6-OHDA and other PD-mimetics have made PC12 cells as a suitable model for the study of PD (12). 

Although some clinical studies have examined the effects of NSAIDs on PD, the findings are controversial. There are several probabilities for such different outcomes, and *in vivo* and *in vitro* studies may warrant the final outcomes. In clinical studies, NSAIDs have been examined as a single category. There is not any comprehensive study on comparison among the protective effects of different classes of NSAIDs. Also, meta-analysis argued about the putative activity of NSAIDs and suggests further clinical and mechanistic researches. Since NSAIDs are widely used as over-the-counter (OTC) drugs without prescription. the results of clinical studies may be conflicting, which leads to false-negative result. So, meta-analysis stated that the results of clinical studies are controversial due to differences in the age, sex, smoking, environmental and nutritional status (13, 14).

Given the characteristics of different NSAIDs in selective inhibition of COX isoforms, it is assumed that specific class of NSAIDs may differ in protective activity. The main aim of this study was to evaluate the pathways which could be altered by NSAIDs in PC12 cell damage by 6-OHDA as* in vitro* model of PD. Furthermore, the antioxidant and anti-apoptotic effects of three different classes of NSAIDs were aimed to be questioned in the present study because of their different and selective inhibition abilities for COX-1 and COX-2. For this purpose, the effect of celecoxib (COX-2 inhibitor), indomethacin (more than 50% selectivity for COX-1) and ibuprofen (COX-1 and COX-2 inhibitors) were compared on cell viability, glutathione (GSH) levels, ROS levels and apoptosis. The expression levels and amount of the main apoptosis biomarker were measured to answer whether the protective effects of NSAIDs can be associated with selective inhibition of NSAIDs on COX-1 and COX-2.

## Materials and Methods

***Reagents and chemicals***

The celecoxib, indomethacin and ibuprofen were purchased from school of pharmacy (Mashhad University of Medical Sciences, Mashhad, Iran); 6-OHDA, the fluorescent probe propidium iodide (PI), Triton X-100, AlamarBlue (resazurin), DCFH-DA (2′, 7′-Dichlorofluorescin diacetate) and DTNB, 5-5’-dithiobis (2-nitrobenzoic acid) were from Sigma (St Louis, MO, USA); RPMI-1640 and fetal bovine serum (FBS) from Gibco (Grand Island, USA); β-actin, caspase-3, Poly (ADP-ribose) polymerase (PARP), stress-activated protein kinases / c-Jun N-terminal kinases (SAPK/JNK), phosphorylated (P)-SAPK/JNK, nuclear factor kappa B (NFκB), Bax and bcl-2 antibodies, anti-rabbit IgG, and horseradish peroxidase (HRP)-linked antibody were from Cell Signaling technology (Boston, USA); ECL Western blotting detection reagent was from Bio-Rad (USA); phosphatase inhibitor cocktail, sodium citrate, phenylmethylsulfonyl fluoride, and Bio-Rad Protein Assay Kit (Hercules, CA, USA) were used; RNA extraction Kit, cDNA synthesis Kit and real-time PCR Kit were from Yektatajhiz (Tehran, Iran). 

***Cell culture***

PC12 cell lines (code numbers: C153) were obtained from Cell Bank at the Pasteur Institute (Tehran, Iran) and were cultured in RPMI 1640 medium supplemented with 10% (v/v) FBS, 100 U/ml penicillium and 100 mg/ml streptomycin. Cells were kept at 37°C in a humidified atmosphere (90%) containing 5% CO_2_. Neuronal differentiation was induced with nerve growth factor (NGF; 100 ng/ml). For each concentration and time course study, there was a control sample, which remained untreated and received an equal volume of the culture medium (15).

***Cell viability analysis***

Resazurin is a cell viability detector, which is using the reducing power of living cells to quantify the proliferation of cells, bacteria, plant and fungi that allow measuring cytotoxicity of various chemicals (16). At first in this step, the cytotoxicity of 6-OHDA (50 - 400 μM) and each one of the celecoxib, indomethacin and ibuprofen (2.5 - 100 μM) was measured in cultures of PC12 cells, then based on results the effects of celecoxib, indomethacin and ibuprofen on 6-OHDA-induced cytotoxicity in PC12 cells were examined. For indicating cell viability, PC12 cells (4×10^3^ cells per well) were seeded in 96-well plates in 100 μl of culture medium and differentiated by NGF (100 nM) for 2 days. Cells were incubated with the different concentration of each one of the compounds (2.5-100 μM) for 24 hr before exposure to 200 μM 6-OHDA. After 24 hr, the Resazurin reagent (20 μl; 10 mg/ml) was added to each well and incubated for 4 hr. The absorbance in 600 nm was measured by ELISA microplate reader and compared with the related control (Awareness, Palm City, FL, USA) (17).

***Intracellular ROS analysis***

DCFH-DA reagent is a cell-permeable non-fluorescent probe, which is used to detect oxidative products in various cells (18). PC12 cells (4×10^3^ cells per well) were cultured in 96-well plates and differentiated with NGF (100 nM) for 2 days. Cells were incubated with selected concentration of each one of compound, which was determined by dose-response curves (2.5 and 5 μM) for 24 hr before exposure to 200 μM 6-OHDA regarding cell viability. After an additional 24 hr, cells were incubated with 2.5 μM DCFH-DA for 30 min. Then, ROS generation was measured by microplate fluorometer and compared with the related control after assessment (excitation wave length, 485 nm; emission wave length, 530 nm) (Paradigm Multi- Mode plate reader; Becton, Dickinson and Company, Franklin Lakes, NJ, USA) (19).

***Intracellular GSH analysis***

DTNB (DTNB, Ellman’s Reagent) reagent is used to measure the concentration of GSH in a variety of animal and plant samples via reacting with GSH to form the 412 nm chromophore, TNB and GS-TNB (20). PC12 cells (10^6^ cells per well) were grown in 6-well plates and differentiated with NGF (100 nM) for 2 days. Cells were incubated with 2.5 and 5 μM of each one of celecoxib, indomethacin and ibuprofen for 24 hr before exposure to 200 μM 6-OHDA. After 4 hr, the DTNB reagent (500 μl; 4 mg/ml) was added to each well. Then, GS-TNB generation was measured with a spectrophotometer at 412 nm and compared with the related control (Jenway 6105 UV/vis, Jenway, UK) (19).

***Flow cytometry analysis of apoptosis***

Flow cytometry and PI staining of treated cells to detect a sub-G1 peak were performed to evaluate the amount of apoptotic cells (21). PC12 cells (2×10^5^ cells per well) were cultured and differentiated with NGF (100 nM) in 12-well plates for 2 days. Cells were treated with 2.5 and 5 μM of each one of celecoxib, indomethacin and ibuprofen for 24 hr before exposure to 200 μM 6-OHDA, followed by an additional 4 hr of incubation. Cells were washed with phosphate-buffer saline (PBS), harvested and incubated at 4°C in the dark with 400 μl of hypotonic buffer (50 μg/ml PI in 0.1% sodium citrate and 0.1% Triton X-100) for 30 sec before flow cytometry analysis (BD Biosciences, CA, USA) (17).

***Real-time PCR analysis***

Real-time PCR analysis was conducted to determine the expression of Bax and Bcl2 gene. Briefly, PC12 cells (10^6^ cells/T25 flask) were grown and differentiated. Then, cells were treated with 2.5 and 5 μM of each one of celecoxib, indomethacin and ibuprofen for 24 hr before exposure to 200 μM 6-OHDA. After 4 hr incubation, cells were washed with cool PBS and harvested. RNA extraction, cDNA synthesis and real-time PCR were preformed according to the manufacturer’s instructions (Yektatajhiz, Tehran, Iran). Primers were designed based on known cDNA sequences (22). The Bax primer: 5’ TGCTGATGGCAACTTCAACT 3’ (forward) and 5’ ATGATGGTTCTGATCAGCTCG 3’ (reverse); the Bcl2 primer: 5’ GGTGGAGGAACTCTTCAGGGA 3’ (forward) and 5’ GGTTCAGGTACTCAGTCATCCA 3’ (reverse); the β-actin primer: 5’ GGGAAATCGTGCGTGACATT 3’ (forward) and 5’ GCGGCAGTGGCCATCTC 3’ (reverse). The RT-generated DNA was amplified using SYBR Green qPCR Master Mix 2X. Finally, to normalize the gene expression levels, β-actin was used as housekeeping gene (Step one thermal cycler, Applied Biosystem, USA) (23).

***Western blotting analysis***

According to the protocol of previous study (17), western blot analysis was used to determine the expression of Bax, Bcl2, caspase-3, PARP, SPAK/JNKs, the active form of SPAK/JNK (P-SPAK/JNK), and NFκB proteins. PC12 cells (10^6^ cells/T25 flask) were grown and differentiated. Then, cells were treated with 2.5 and 5 μM of each one of celecoxib, indomethacin and ibuprofen for 24 hr before exposure to 200 μM 6-OHDA. Followed by an additional 4 hr of incubation, cells were washed with cool PBS and harvested. Finally, the expression levels of these proteins were normalized with respect to β-actin (Gel Doc UV Alliance, Alliance 4.7, UK).

***Statistics analysis***

One-way ANOVA followed by Tukey-Kramer *post hoc* test was used for comparing differences between groups and two-way ANOVA for comparing differences between NSAIDs. All results were presented as mean ± SD and *P- *values below <0.05 was regarded statistically significant. Each experiment was repeated at least three times.

## Results

***Effects of celecoxib, indomethacin and ibuprofen on cytotoxicity induced by 6-OHDA in PC12 cells***

**Figure 1:**
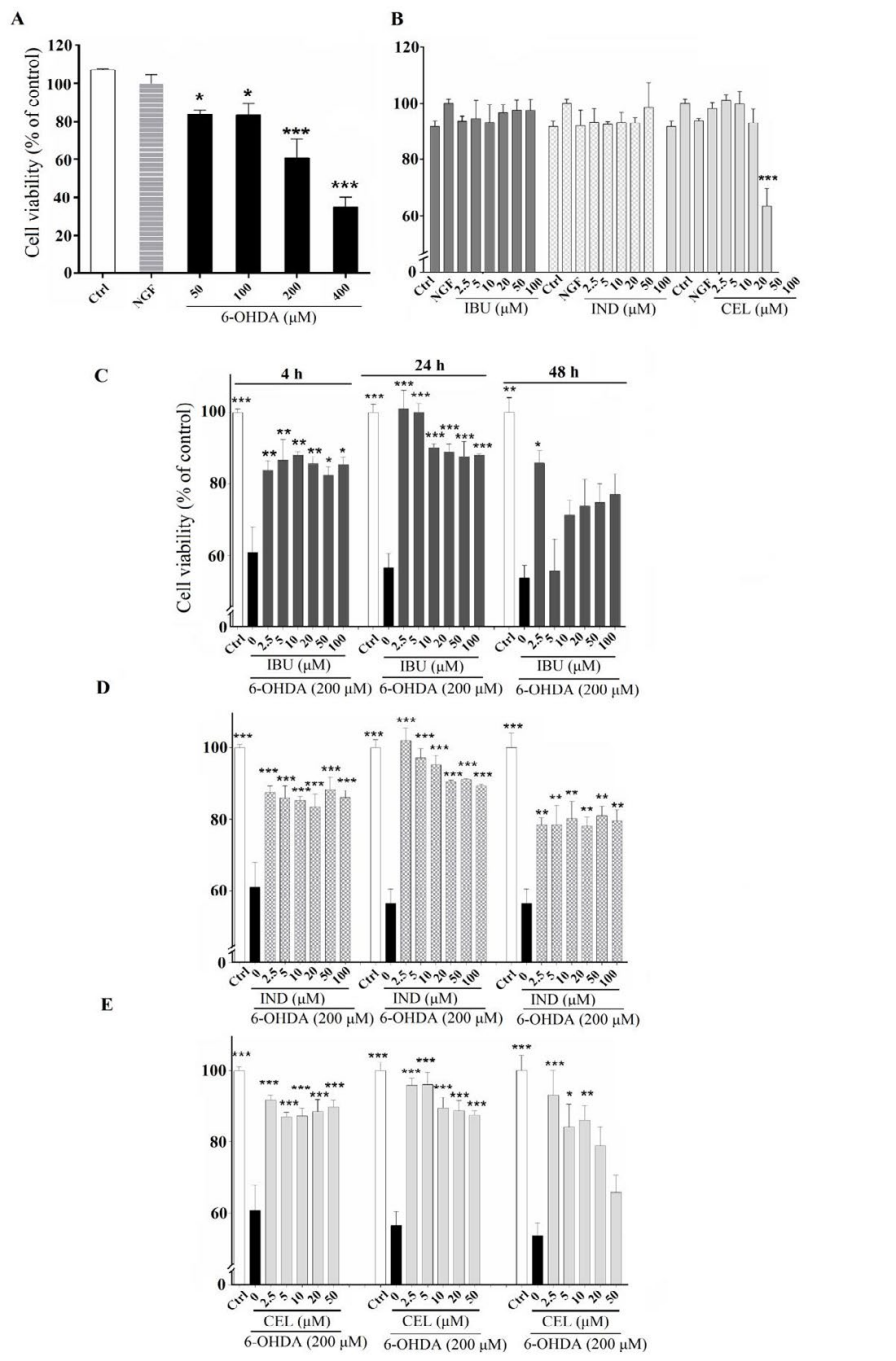
Cell viability: (A) Effects of various concentrations (50- 400 μM) of 6-hydroxydopamine (6-OHDA) on the viability of PC12 cells differentiated by nerve growth factor (NGF). (B) Effects of various concentrations (2.5 - 100 μM) of celecoxib, indomethacin and ibuprofen on the viability of NGF-differentiated PC12 cells. (C) Effects of various concentrations (2.5 - 100 μM) of ibuprofen in NGF-differentiated PC12 cells induced by 6-OHDA. (D) Effects of various concentrations (2.5 - 100 μM) of indomethacin in NGF-differentiated PC12 cells induced by 6-OHDA. (E) Effects of various concentrations (2.5 - 100 μM) of celecoxib in NGF-differentiated PC12 cells induced by 6-OHDA. Values are the mean±SD. *P<0.05, **P<0.01 and ***P< 0.001 compared to 6-OHDA group. All experiments were performed in triplicate (at least 3 independent experiments into each of four wells of a 96-well plate)

The optimum cytotoxic concentration (CC) of 6-OHDA that causes 50% cytotoxic effect (CC_50_) was chosen based on the literatures (19, 24, 25). Moreover, the protective activity of NSAIDs against 6-OHDA could be well differentiated using the CC50 (8). According to our results, when PC12 cells were incubated with 200 μM of 6-OHDA for 24 hr, cell viability significantly decreased by 60±9.8% compared to untreated cells (*P<*0.001) (Figure 1A). The cytotoxicity of celecoxib, indomethacin and ibuprofen were examined before evaluating their effects on 6-OHDA-induced cytotoxicity in PC12 cells. Cell viability of PC12 cells has shown no significant changes compared to untreated cells when were incubated with different concentrations of each one of celecoxib (2.5 -50 μM), indomethacin and ibuprofen (2.5 -100 μM) for 24 hr (Figure 1B). All of three NSAIDs compounds significantly inhibited 6-OHDA-induced cytotoxicity when PC12 cells were treated with test compounds (2.5-100 μM) 4, 24 and 48 hr before 6-OHDA exposure. The protective effects of these compounds were concentration and time-dependent, so a concentration two time lower than the inhibitory concentration (IC_50_) values for each compounds and pretreatment time of 24 hr was selected, because of the best action of the NSAIDs compounds were appeared in this time point for performing the subsequent experiments (Figure 1C, 1D, 1E).

**Figure 2:**
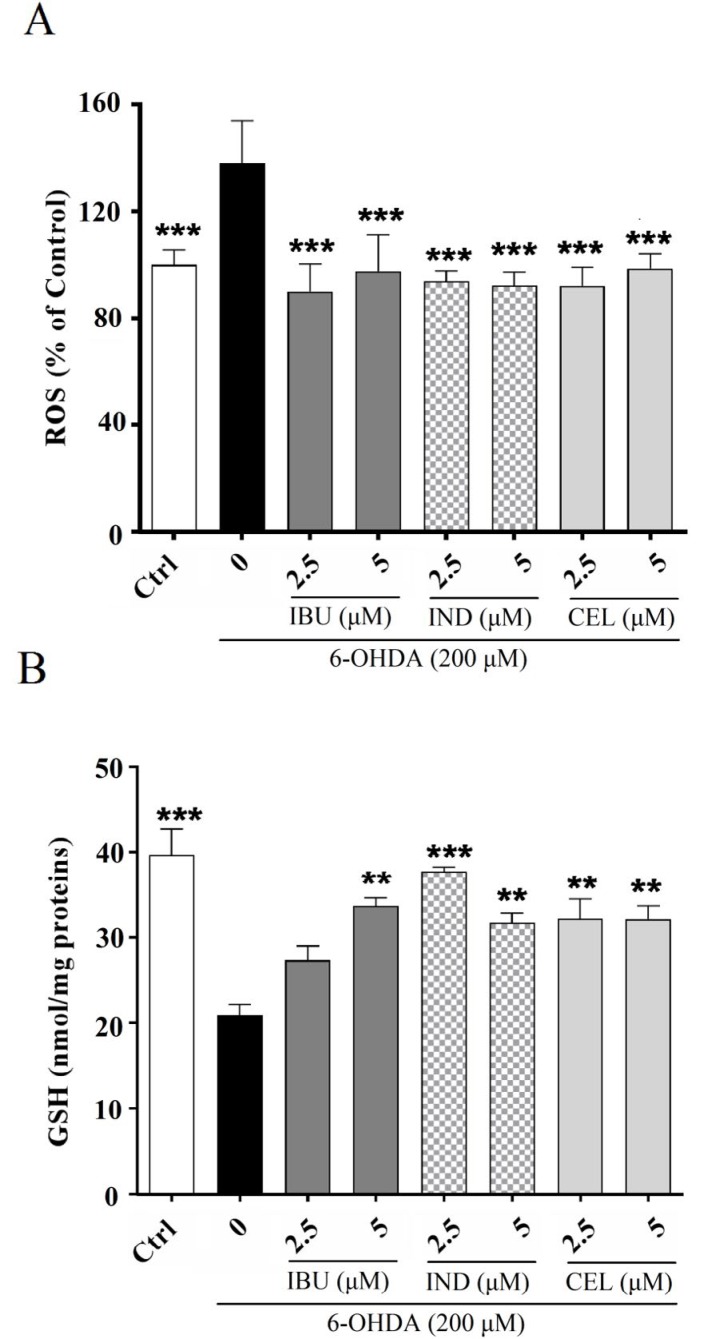
Effects of celecoxib, indomethacin and ibuprofen (2.5 and 5 μM) on intracellular (A) reactive oxygen species (ROS) and (B) glutathione (GSH) in nerve growth factor (NGF)-differentiated PC12 cells induced by 6-hydroxydopamine (6-OHDA). (A) All three compounds (celecoxib, indomethacin and ibuprofen) could prevent 6-OHDA-induced ROS increment. (B) All three compounds could prevent 6-OHDA-induced GSH reduction. Values are the mean ± SD. *P< 0.05, **P< 0.01 and ***P< 0.001 compared to 6-OHDA group. All experiments were performed in triplicate ((A) at least 3 independent experiments into each of four wells of a 96-well plate, (B) at least 3 independent experiments into each of three wells of a 6-well plate)

**Figure 3:**
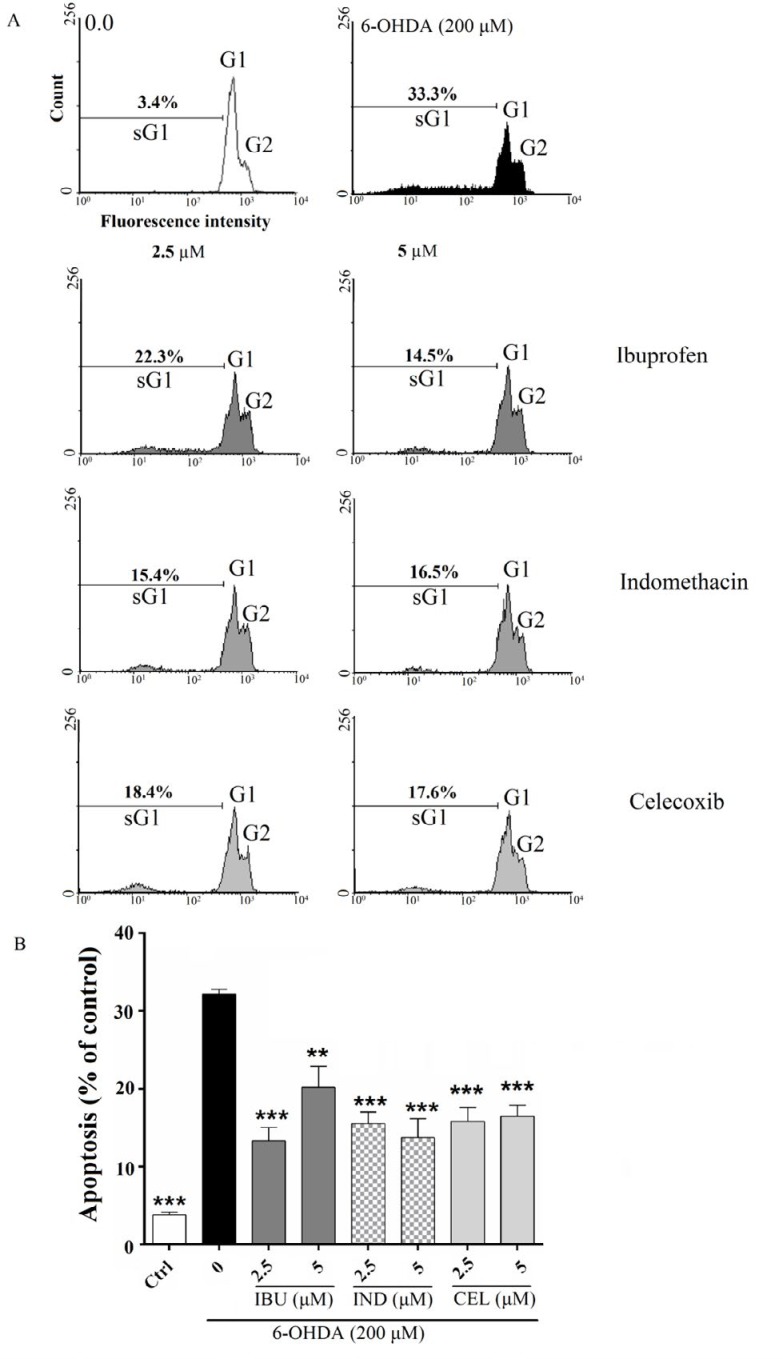
Effects of celecoxib, indomethacin and ibuprofen (2.5 and 5 μM) on apoptosis by flowcytometry with propidium iodide (PI) in nerve growth factor (NGF)-differentiated PC12 induced by 6-hydroxydopamine (6- OHDA). (A) and (B) pretreatment of the cell with celecoxib, indomethacin and ibuprofen significantly prevented 6-OHDA-induced apoptosis. Values are the mean±SD. *P<0.05, **P<0.01 and ***P<0.001 compared to 6-OHDA group. All experiments were performed in triplicate (at least 3 independent experiments into each of three wells of a 12-well plate)

**Figure 4:**
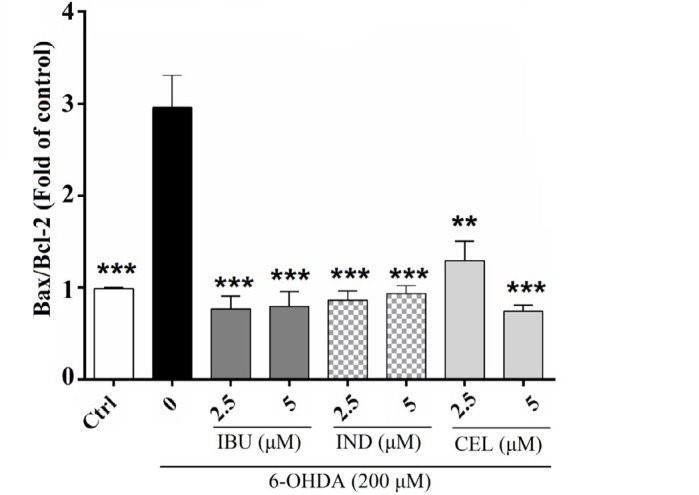
Effects of celecoxib, indomethacin and ibuprofen (2.5 and 5 μM) on Bax/Bcl-2 expression by real-time PCR analysis in nerve growth factor (NGF)-differentiated PC12 cells induced by 6-hydroxydopamine (6-OHDA). Cell pretreatment with celecoxib, indomethacin and ibuprofen decreased Bax/Bcl-2 to a level near that of control and the Bax/Bcl-2 ratio is slightly similar for all three drugs. Values are the mean±SD. *P<0.05, **P<0.01 and ***P<0.001 compared to 6-OHDA group. All experiments were performed in triplicate

**Figure 5:**
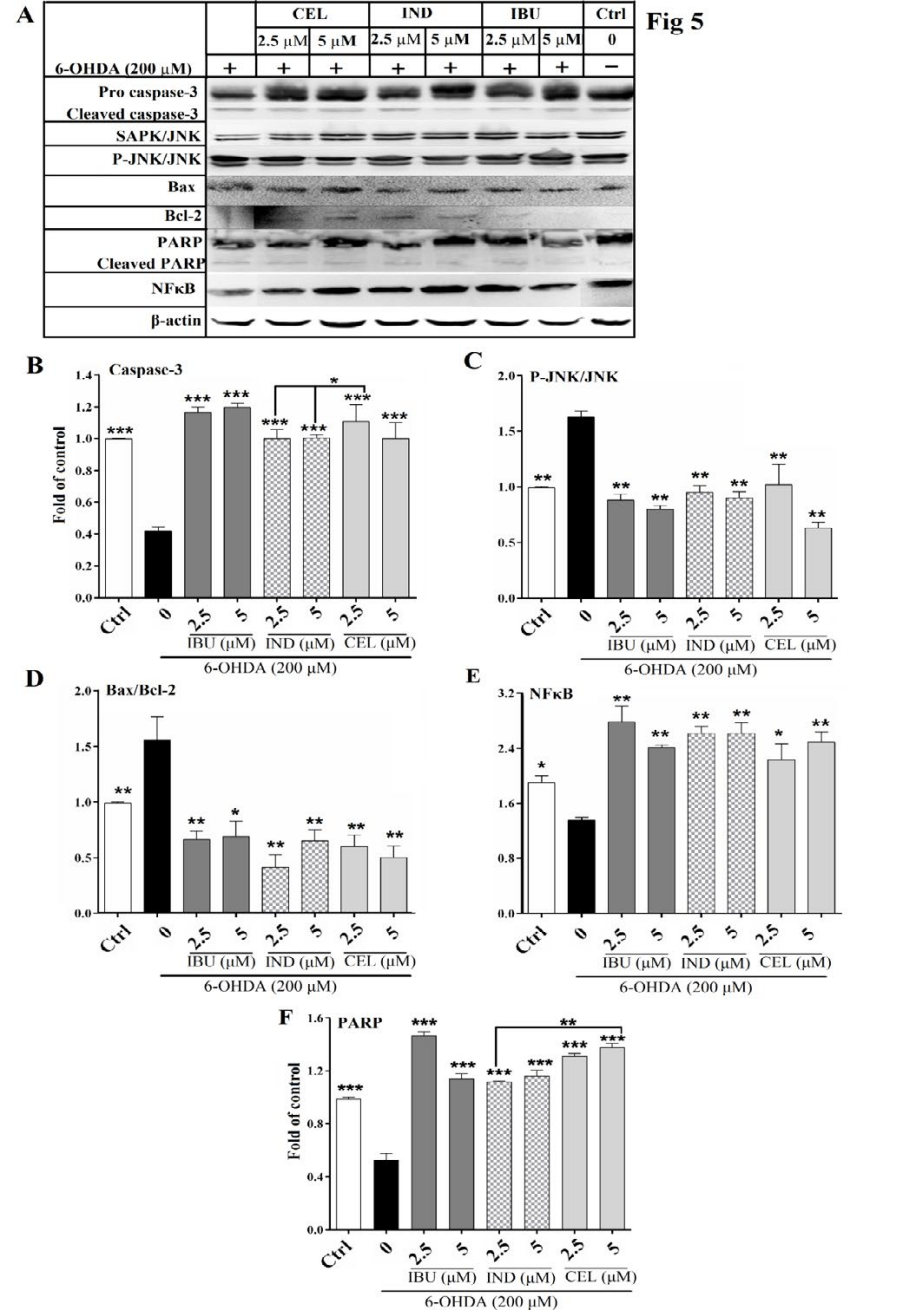
Effects of celecoxib, indomethacin and ibuprofen (2.5 and 5 μM) on protein levels of caspase-3, Bax, Bcl-2, stress-activated protein kinases/c-Jun N-terminal kinases (SAPK/JNK), P-SPAK/JNK, poly (ADP-ribose) polymerase (PARP), and nuclear factor kappa B (NFκB) by western blot analysis in nerve growth factor (NGF)-differentiated PC12 cells induced by 6-hydroxydopamine (6-OHDA). Cell pretreatment with celecoxib, indomethacin and ibuprofen decreased (A) The western blotting image (B) caspase-3 (*P*<0.001), (C) P-SPAK/JNK/SAPK/JNK (*P*<0.01), (D) Bax/Bcl-2 (*P*<0.01) and (E) increased cytoplasmic content of NFκB (*P*<0.01) and (F) decreased cleaved PARP (*P*<0.001) levels to a level near that of control. Values are the mean±SD. *P<0.05, ** *P*<0.01 and *** *P*<0.001 compared to 6-OHDA group. All experiments were performed in triplicate

***Effects of celecoxib, indomethacin and ibuprofen on 6-OHDA-induced ROS in PC12 cells***

It is known that the primary source of 6-OHDA-induced cytotoxicity is ROS production generated via 6-OHDA oxidation (19). The results of current study also confirm these reports, because intracellular ROS levels significantly increased when PC12 cells were induced by 6-OHDA. The results of this research indicated that cell pretreatment with celecoxib, indomethacin and ibuprofen (2.5 and 5 μM) significantly suppressed ROS generation and showed a protective effect against 6-OHDA-induced cytotoxicity (Figure 2A).

***Effects of celecoxib, indomethacin and ibuprofen on GSH levels in 6-OHDA-treated PC12 cells***

In previous studies, it has been found that GSH levels were reduced in a rat model of PD, which might be secondary to the ROS production induced by 6-OHDA (24). Based on the results of the present study, GSH levels were 21±2.09 (nmol/mg proteins) in 6-OHDA-treated PC12 cells compared to untreated cells with 39.7±5.27 (nmol/mg proteins) GSH levels (*P<*0.001). When PC12 cells were treated with celecoxib, indomethacin and ibuprofen (2.5 and 5 μM) 24 hr before exposure to 6-OHDA, GSH levels significantly increased, which means that all three compounds could prevent the oxidation induced by 6-OHDA (Figure 2B).

***Effects of celecoxib, indomethacin and ibuprofen on 6-OHDA-induced cell death in PC12 cells based on flow cytometry analysis***

In previous studies, it has been indicated that 6-OHDA induces apoptosis in PC12 (19). The results also confirmed 6-OHDA induced apoptosis, because when PC12 cells were exposed to 6-OHDA, the percentage of apoptotic cells were 31.7±2.3% compared to untreated cells with 3.4±1.1% apoptotic cells (*P<*0.001). Cell pretreatment with celecoxib, indomethacin and ibuprofen (2.5 and 5 μM) significantly prevented 6-OHDA-induced apoptosis (Figure 3).

***Effects of celecoxib, indomethacin and ibuprofen on 6-OHDA-induced cell death in PC12 cells based on real-time PCR analysis***

It has been found that, anti-apoptotic Bcl-2 family proteins could prevent the apoptotic effect of Bax through inhibiting the incorporation of Bax to Bax channel on a mitochondrial membrane (25). In this study, in incubated PC12 cells with 6-OHDA (200 μM), the expression of Bax/Bcl2 was 2.9±0.49% compared to untreated cells with 0.99±0.01% (*P<*0.001). In contrast, pretreatment with celecoxib, indomethacin and ibuprofen (2.5 and 5 μM) in PC12 cells treated with 6-OHDA decreased Bax/Bcl-2 to a level similar to the related control (Figure 4).

***Effects of celecoxib, indomethacin and ibuprofen on 6-OHDA-induced cell death in PC12 cells based on western blot analysis***

Recent studies suggested that 6-OHDA activates cellular apoptosis pathways through stimulation of some main modulators of apoptosis (25). But, it has not been determined whether celecoxib, indomethacin and ibuprofen could inhibit 6-OHDA-induced apoptotic cell death and which modulators of 6-OHDA-induced apoptosis pathways are suppressed in NSAIDs-treated PC12 cells. In the present study, pretreatment with celecoxib, indomethacin and ibuprofen (2.5 and 5 μM) in PC12 cells treated with 6-OHDA decreased caspase-3 (*P<*0.001), Bax/Bcl-2 (*P<*0.01), P-SPAK/JNK/SAPK/JNK (*P<*0.01) and cleaved PARP (*P<*0.001) and increased cytoplasmic content of NFκB (*P<*0.01) levels to a level near that of control. Two-way ANOVA analysis showed that celecoxib was significantly more potent than indomethacin in protection against 6-OHDA-induced apoptosis shown by protection against caspase-3 (*P<*0.05) and PARP cleavage (*P<*0.01) (Figure 5).

## Discussion

PD is known as the second most common neurodegenerative disorder after Alzheimer’s disease; however, its etiology is still not clarified (25). Multiple animal studies (*in vivo* studies) have examined the effects of NSAIDs on PDbut the molecular mechanism has not been well presented. Also, the anti-apoptotic effects of NSAIDs have not been addressed comprehensively (26, 27). The detailed mechanism of 6-OHDA as one of the main important neurotoxin in PDhas not been well addressed in the literature. Here, we have examined the possible protective mechanism of three different classes of NSAIDs against 6-OHDA. The pathways of SAPK/JNK and NFkB were the main targets of the present study (25). 

Besides conventional treatment, recent advances show that patient may benefit from NSAIDs therapy (4). Here we have searched for the mechanism(s) by which NSAIDs may exert protective effects against 6-OHDA-induced cell death in PC12 cells as an accepted *in vitro* model of PD (12). According to the results, pre-treatment with celecoxib, indomethacin and ibuprofen (2.5 and 5 μM) in PC12 cells treated with 6-OHDA showed a significant increase in cell viability, intracellular GSH levels and decrease in the amount of ROS and apoptosis. Following treatment with NSAIDs, ROS production was significantly decreased, which suggests that the protective effects of celecoxib, indomethacin and ibuprofen may be mediated via ROS scavenging property. Protection against PD progression has been referenced for NSAIDs and recently the antioxidant effects have been suggested as the possible mechanism (28) as also it happened in the present study. Decrease in the amount of ROS and consequently cell death followed by treatment with NSAIDs can confirm the antioxidant effect of NSAIDs. Swiatkiewicz and colleagues (2013) reported that ibuprofen protects against ROS increment followed by mitochondria dysfunction and then the death of dopaminergic neuron via decreasing dopamine turnover and COX inhibition in MPTP (1-methyl-4-phenyl-1,2,3,6-tetrahydropyridine)-injured mice (29). Two review articles published in 2010 concluded that NSAIDs exhibit neuroprotective effects in neurodegenerative diseases, including PD through scavenging ROS (28, 4). Končič and colleagues (2009) reported that fenoprofen, ketoprofen, indomethacin, ibuprofen, and diclofenac showed significant antioxidant effects via decreasing ROS compared to butylated hydroxyanisole using a β-carotene-linoleic acid model system (30). Similar to our findings, decrease in ROS has been suggested as the mechanism for protection against neural cell death with NSAIDs. 

Also, we examined if NSAIDs could be able to protect against apoptosis induced by 6-OHDA. For this purpose, the expression and the level of some of the main apoptosis mediators such as Bax, Bcl-2, caspase-3, PARP, SPAK/JNK, the active form of SPAK/JNK, and cytoplasmic content of NFκB proteins were measured in cells. Pre-treatment with celecoxib, indomethacin and ibuprofen (2.5 and 5 μM) in PC12 cells treated with 6-OHDA could lead to decrease in 6-OHDA cytotoxicity through suppressing the activation of nuclear content of NFκB and SPAK/JNK. Activation of caspase-3 is associated with apoptosis. Numerous recent finding showed that the activation of caspase-3 is induced by 6-OHDA following ROS production, then change in conformation of Bcl-2 promotes cell to apoptosis. Bcl-2 inhibits the loss of mitochondrial membrane potential and cell death induced by Bax. Also, activated caspase-3 cleaves a number of structural proteins such as PARP, whose cleavage is one of the main signs of cell destruction (25). The SAPK/JNKs are a group of MAPK (mitogen-activated protein kinases), which could mediate apoptotic cell death. Previous studies determined a rise in the active form of JNK (P-SAPK/JNK) by 6-OHDA-induced ROS production (25). NFκB is a transcription factor, which is a member of Rel-family and exists in an inactive form (IκB- NFκB) in the cytoplasm. Previous studies detected that following oxidative stress caused by 6-OHDA, IκB is phosphorylated, which leads to increasing nuclear content of NFκB (decreasing cytoplasmic content of NFκB) and binding of NFκB to specific DNA sequences. Then, NFκB activates the transcription of genes, which promote the cell death (25).

Only a few studies suggested the protective effect of NSAIDs against apoptotic cell death as the possible mechanism of cell injury induced by 6 OHDA. Bassani and colleagues in a review article (2014) reported that the possible mechanism for protective effects of ibuprofen is associated with reduction in NFκB, COX-2 and inducible nitric oxide synthase (iNOS) expression and reactive nitrogen species (RNS) production through the binding to the anti-inflammatory peroxisome proliferator-activated receptor γ (PPARγ) (31). In another study published in 2010, authors reported that NSAIDs exhibit neuroprotective effects via inhibition of NFκB activity, which causes inhibition in the transcription of target genes. Also, the suppression of P38 activity has been suggested as the protective effects of NSAIDs such as aspirin (ASA), ibuprofen, and NS-398 (4). Similarly, Asanuma and colleagues (2008) have concluded that NSAIDs scavenge RNS by inhibiting NFκB activity in experimental parkinsonian models and PD (32). In a study, NOSH-ASA (NBS- 1120), as a NO- and H_2_S-releasing hybrid of aspirin, showed the protective effects against toxins that are released by activated microglia and astrocytes. This protection happened via a decreasing in the release of tumor necrosis factor alpha (TNFα) and interleukin 6 (IL-6) and activity of P38 and NFκB in comparison with S-ASA or NO-ASA. So the authors suggested a consideration for NOSH-ASA as an appropriate combination for the treatment of neurodegenerative diseases, including PD (33). In 2011, Gupta and colleagues examined the effect of COX-2 inhibitors (valdecoxib or NS-398) on a MPTP-induced animal model of PD and reported the protective role of COX-2 inhibitors through a reduction in caspase-3 and NFκB activity (26).

Like many other studies, we found that COX-2 inhibitor NSAIDs are more effective in protection against apoptosis than COX-1 inhibitor NSAIDs. Increasing the expression of COX-2 is associated with chronic inflammatory diseases such as rheumatoid arthritis and neurological disorders such as PD (34). This finding suggested that suppressing NFκB protein level by celecoxib may reduce binding of NFκB to COX-2 promotors and COX-2 mRNA expression, which are associated with inhibition of PGE_2_ and PGI_2_ synthesis and anti-inflammatory response (5, 35). On the other hand, COX-2 induces and mediates inflammatory responses, which finally increase oxidative reactions, ROS generation, and ultimately apoptosis (9). It has been reported that the use of COX-2 inhibitor NSAIDs can effectively scavenge ROS and possess antioxidant effects and also reduce apoptosis by suppressing SAPK/JNK pathway, while the COX-1 inhibitor NSAIDs do not show the same action. On the other hand, the severe gastrointestinal complications of NSAIDs are related to the inhibition of COX-1. To sum up, COX-2 inhibitors NSAIDs have been introduced as anti-apoptotic agents with less adverse effect (36, 30). 

## Conclusion

It is suggested that pretreatment with celecoxib, indomethacin and ibuprofen would probably reduce the cellular injuries induced by 6-OHDA as an acceptable *in vitro* model of PD. The NFκB and SAPK/JNK pathways seems to have an important role in cell injury induced by 6-OHDA and have been suggested as the possible mechanism of apoptosis for *in vitro* model of PD. NSAIDs effectively protected against activation of NFκB and SAPK/JNK pathways by 6-OHDA.

Interestingly, celecoxib was more potent in protection against 6-OHDA-induced apoptosis than indomethacin, which was shown by different activity in protection against caspase-3 and PARP cleavage. In general, according to the reported results of the previous studies and the results of the present study, it seems that COX-2 inhibitors may mediate more protective effect against 6-OHDA-induced apoptosis than COX-1 inhibitors.
